# Oncolytic activity of reovirus in HPV positive and negative head and neck squamous cell carcinoma

**DOI:** 10.1186/s40463-015-0062-x

**Published:** 2015-02-24

**Authors:** Timothy Cooper, Vincent L Biron, David Fast, Raymond Tam, Thomas Carey, Maya Shmulevitz, Hadi Seikaly

**Affiliations:** Division of Otolaryngology - Head and Neck Surgery, Department of Surgery, University of Alberta, 1E4 University of Alberta Hospital, 1E4 Walter Mackenzie Center, 8440 112 St., Edmonton, AB T6G 2B7 Canada; Faculty of Science 1–001 CCIS, University of Alberta, Edmonton, AB T6G 2E9 Canada; Faculty of Medicine and Dentistry, University of Alberta, 2J2 WC Mackenzie Health Sciences Centre, Edmonton, AB T6G 2R7 Canada; Department of Head and Neck Surgery, University of Michigan, 5311B Med Sci I, Ann Arbor, MI 48109-5616 USA; Department of Medical Microbiology and Immunology, University of Alberta, 6–142 J Katz Group Centre for Pharmacy & Health Research, Edmonton, AB T6G 2E1 Canada

**Keywords:** Reovirus, Head and neck cancer, Squamous cell carcinoma, HPV

## Abstract

**Background:**

The management of patients with advanced stages of head and neck cancer requires a multidisciplinary and multimodality treatment approach which includes a combination of surgery, radiation, and chemotherapy. These toxic treatment protocols have significantly improved survival outcomes in a distinct population of human papillomavirus (HPV) associated oropharyngeal cancer. HPV negative head and neck squamous cell carcinoma (HNSCC) remains a challenge to treat because there is only a modest improvement in survival with the present treatment regimens, requiring innovative and new treatment approaches. Oncolytic viruses used as low toxicity adjunct cancer therapies are novel, potentially effective treatments for HNSCC. One such oncolytic virus is Respiratory Orphan Enteric virus or reovirus. Susceptibility of HNSCC cells towards reovirus infection and reovirus-induced cell death has been previously demonstrated but has not been compared in HPV positive and negative HNSCC cell lines.

**Objectives:**

To compare the infectivity and oncolytic activity of reovirus in HPV positive and negative HNSCC cell lines.

**Methods:**

Seven HNSCC cell lines were infected with serial dilutions of reovirus. Two cell lines (UM-SCC-47 and UM-SCC-104) were positive for type 16 HPV. Infectivity was measured using a cell-based ELISA assay 18 h after infection. Oncolytic activity was determined using an alamar blue viability assay 96 h after infection. Non-linear regression models were used to calculate the amounts of virus required to infect and to cause cell death in 50% of a given cell line (EC_50_). EC_50_ values were compared.

**Results:**

HPV negative cells were more susceptible to viral infection and oncolysis compared to HPV positive cell lines. EC_50_ for infectivity at 18 h ranged from multiplicity of infection (MOI) values (PFU/cell) of 18.6 (SCC-9) to 3133 (UM-SCC 104). EC_50_ for cell death at 96 h ranged from a MOI (PFU/cell) of 1.02×10^2^ (UM-SCC-14A) to 3.19×10^8^ (UM-SCC-47). There was a 3×10^6^ fold difference between the least susceptible cell line (UM-SCC-47) and the most susceptible line (UM-SCC 14A) EC_50_ for cell death at 96 h.

**Conclusions:**

HPV negative HNSCC cell lines appear to demonstrate greater reovirus infectivity and virus-mediated oncolysis compared to HPV positive HNSCC. Reovirus shows promise as a novel therapy in HNSCC, and may be of particular benefit in HPV negative patients.

## Background

Head and neck squamous cell carcinoma (HNSCC) is a devastating disease that affects all aspects of the patient’s life, even in survivorship [1]. The management of patients with advanced stages of this disease requires a multidisciplinary and multimodality treatment approach which includes a combination of surgery, radiation, and chemotherapy. These toxic treatment protocols have significantly improved survival outcomes, especially in a distinct population of human papillomavirus (HPV) associated oropharyngeal cancer [2-7]. HPV is an important risk factor for a subset of HNSCC [8-10] and types 16 and 18 are particularly high risk for oncogenic transformation [11]. Patients with HPV associated head and neck cancer tend to be younger and less likely to have a significant history of smoking and alcohol consumption in comparison to those affected by non-HPV related head and neck cancer [8,12]. Advanced stage HPV negative HNSCC remains a challenge to treat because there is only a modest improvement in survival outcomes despite advances in therapy and the increasing toxicity of the different protocols [2,4-6]. This subset of patients, therefore, requires innovative and new treatment approaches.

The use of oncolytic viruses as a low toxicity adjunct cancer therapy is a novel and potentially effective treatment for HNSCC. One such oncolytic virus is Respiratory Orphan Enteric virus or reovirus [13-18]. Reovirus, from the family *Reoviridae*, is a non-enveloped, double stranded RNA virus that infects the upper respiratory and gastrointestinal tracts of humans with minimal symptoms [19]. Reovirus shows potent anti-tumor activity in a variety of tumor models, including models of HNSCC [20-27]. Multiple mechanisms mediate the strong specificity of reovirus towards cancer cells and especially towards cells with activated Ras signalling [16,28-33]. A proprietary formulation of the type 3 Dearing reovirus strain, called Reolysin®, is undergoing numerous phase I and phase II clinical trials and is currently in a phase III trial [19,34,35].

Susceptibility of HNSCC cells towards reovirus infection and reovirus-induced cell death has been previously demonstrated in both *in vitro* and mouse models [22,26,36,37], but the effectiveness and infectivity of reovirus in HPV positive and negative head and neck cancer cell lines has not been examined. The objectives of this study were to compare the infectivity and oncolysis of reovirus in HPV positive and negative HNSCC cell lines.

## Methods

### Cell lines

SCC-9, SCC-25, FaDU and L929 were purchased from ATC and maintained according to instructions. UM-SCC-14A, UM-SCC-38, UM-SCC-47, and UM-SCC-104 were obtained from Dr. Thomas Carey at the University of Michigan and maintained according to instructions. UM-SCC-47 and UM-SCC-104 are both positive for high risk HPV 16 and express viral proteins E6 and E7 [38-40].

### Virus

Reovirus serotype 3 Dearing was propagated in L929 cells and purified by ultracentrifugation on cesium chloride (CsCl) gradients as previously described [41]. Virus-infected cells were freeze-thawed and twice extracted with Vertrel XF (Dymar Chemicals) as previously described [41] and then layered onto 1.25- to 1.45-g/ml CsCl gradients. Virus was banded at 23,000 rpm for 5 h and dialyzed extensively against virus dilution buffer (150 mM NaCl, 15 mM MgCl_2_, 10 mMTris, pH 7.4). Titers of purified reovirus preparations were obtained using standard plaque titration on L929 cells, and expressed as plaque forming units (PFU) per millilitre [32].

### Seeding and infection of cells

Cells were counted using a TC20 automated cell counter (BioRad). 125 μL of cells at a concentration of 2.5×10^5^ cells/mL were seeded into each well of a 96 well plate to achieve 100% confluence at time of infection. Serial dilutions of reovirus serotype 3 Dearing ranging from 4.8×10^8^ to 1.43×10^1^ PFU/mL (relative to L929 cells) were prepared in minimal essential media (MEM). Cells were incubated with 50 μl of virus at 37°C for 1 hour, then returned to virus-free complete medium for the remaining incubation period under standard tissue culture conditions.

### Cell-based ELISA assay for infectivity

Eighteen hours after infection, cells were washed with PBS, fixed with methanol, and stored in blocking solution (Bovine serum albumin, PBS, Triton X-100). Cells were incubated with rabbit anti-reovirus primary antibody (1:5000, blocking solution), washed with PBS-T (PBS, Triton X-100) solution, then incubated with goat anti-rabbit alkaline phosphatase antibody (1:4000, blocking solution). Following extensive washes with PBS-T, 200 μL of P-nitrophenyl phosphate in diethanolamine buffer (1 mg/mL) was added to each well. Plates were incubated at room temperature for 80 minutes, and absorbance was measured at 405 nm using a spectrophotometer (EnVision Multilabel Reader, Perkin Elmer).

### Alamar blue viability assay

Alamar blue is a commonly used indicator in cell viability assays [42]. At 96 hours after infection, 20 μL of 440 μM alamar blue in sterile PBS diluted 1:10 with ddH_2_O was added to each well of a 96-well plate. Following incubation for 2 hours at 37°C, fluorescence was measured at excitation/emission wavelengths of 544/590 nm respectively (Fluostar OPTIMA plate reader, BMG Labtech).

### Calculation of 96 hour viability

Using the measured fluorescence from the alamar blue assay, viability at 96 h was calculated in the well infected with reovirus at a concentration of 2.40×10^8^ PFU/mL. Fluorescence was averaged from two or more duplicates within each experiment. Viability was expressed as a percentage with 100% viability determined by the fluorescence of the uninfected cells and 0% viability calculated as an average of the fluorescence of wells containing media but not seeded with cells. Mean viability was calculated for each cell line from three or more independent experiments. Statistically significant outliers and experiments with technical issues related to uneven seeding of cells were excluded from analysis.

### Calculation of EC_50_ values

Effective concentration 50 or EC_50_ is a term used in pharmacodynamics indicating the concentration required to have a 50% maximal effect. In the context of infection with a virus, we have defined EC_50_ to indicate the amount of virus needed to infect 50% of cells at 18 hours postinfection, as measured by a cell-based ELISA assay. To quantify reovirus-induced cell death, we have defined EC_50_ to indicate the amount of virus required to reduce cell viability to 50% (relative to untreated cells) at 96 hours postinfection, as measured by an alamar blue viability assay. Absorbance (infectivity) or fluorescence (cell viability) values were plotted against multiplicity of infection (MOI, PFU/cell). Baseline and maximum response were established from uninfected cells (maximum viability, minimum infectivity), media alone (minimum viability), or maximally-infected L929 cells (maximum infectivity). Mean absorbance or fluorescence at a given viral concentration was calculated as the mean of two or more duplicates within the same experiment. Three or more independent experiments were used to generate a dose–response curve for each cell line (Prism; Graph-Pad Software Inc., San Diego, CA). From this, EC_50_ values were calculated by fitting a standard equation for a sigmoidal dose–response curve.

### Statistical analysis

Student’s *t*-test was used to compare EC_50_ values for infectivity and oncolysis between cell lines. Student’s *t*-test was also used to compare cell viability at 96 h. P < 0.05 was accepted as statistically significant.

### Ethics

Institutional ethics review board approval was obtained from the University of Alberta Health Research Ethics Board prior to the commencement of the study.

## Results

### Infectivity

**Figure 1:**
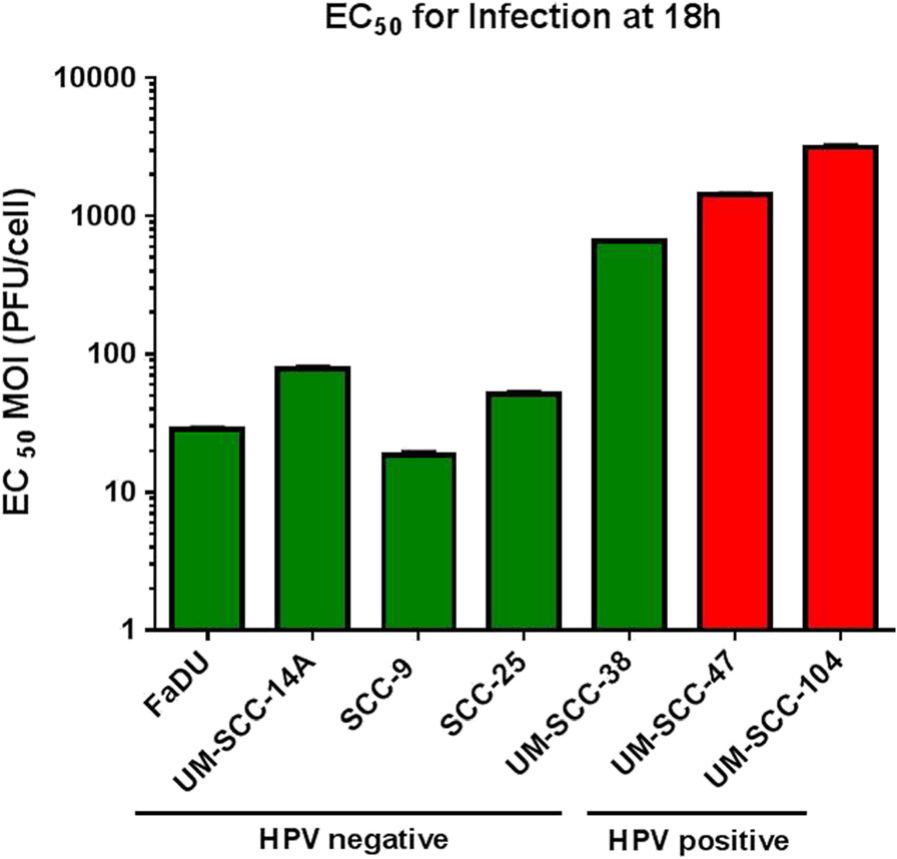
**EC**_**50**_ **values for infection by reovirus after 18 h of various HPV negative and positive HNSCC cell lines.** Error bars represent standard deviation.

EC_50_ MOI for infectivity at 18 h indicates the number of reovirus particles per cell that were sufficient to achieve infection and active replication in 50% of cells at this time point. The HNSCC cell lines demonstrated variable susceptibility to infection by reovirus at 18 h. The cell lines listed from most to least susceptible to reovirus infection at 18 h and their corresponding EC_50_ MOI values (PFU/cell) were SCC-9 (18.6 ± 0.7), FaDU (28.4 ± 0.7), SCC-25 (51.2 ± 1.6), UM-SCC-14A (77.3 ± 3.1), UM-SCC-38 (651 ± 11), UM-SCC-47 (1425 ± 23), and UM-SCC-104 (3133 ± 86) (Figure 1). The most susceptible HNSCC cell lines were SCC-9 and FaDU. These cell lines required a mean of 18.6 and 28.4 virus particles per cell to achieve 50% infectivity at 18 h respectively. The least susceptible cell lines, UM-SCC-47 and UM-SCC-104, were both HPV positive. They required a mean of 1425 and 3133 virus particles per cell to achieve 50% infection, respectively. In comparing the two HPV positive cell lines individually to each of the 5 HPV negative cell lines, the HPV positive HNSCC cell lines were less susceptible to infection by reovirus with statistical significance (p < 0.01).

### 96 h viability

**Figure 2:**
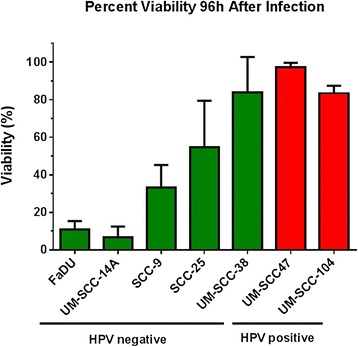
**Percentage of viable cells of various HNSCC cell lines 96 h after addition of 2.40×10** ^**8**^ **PFU/mL dilution of reovirus.** Mean values were taken from three or more independent experiments. Error bars represent standard deviation.

**Figure 3:**
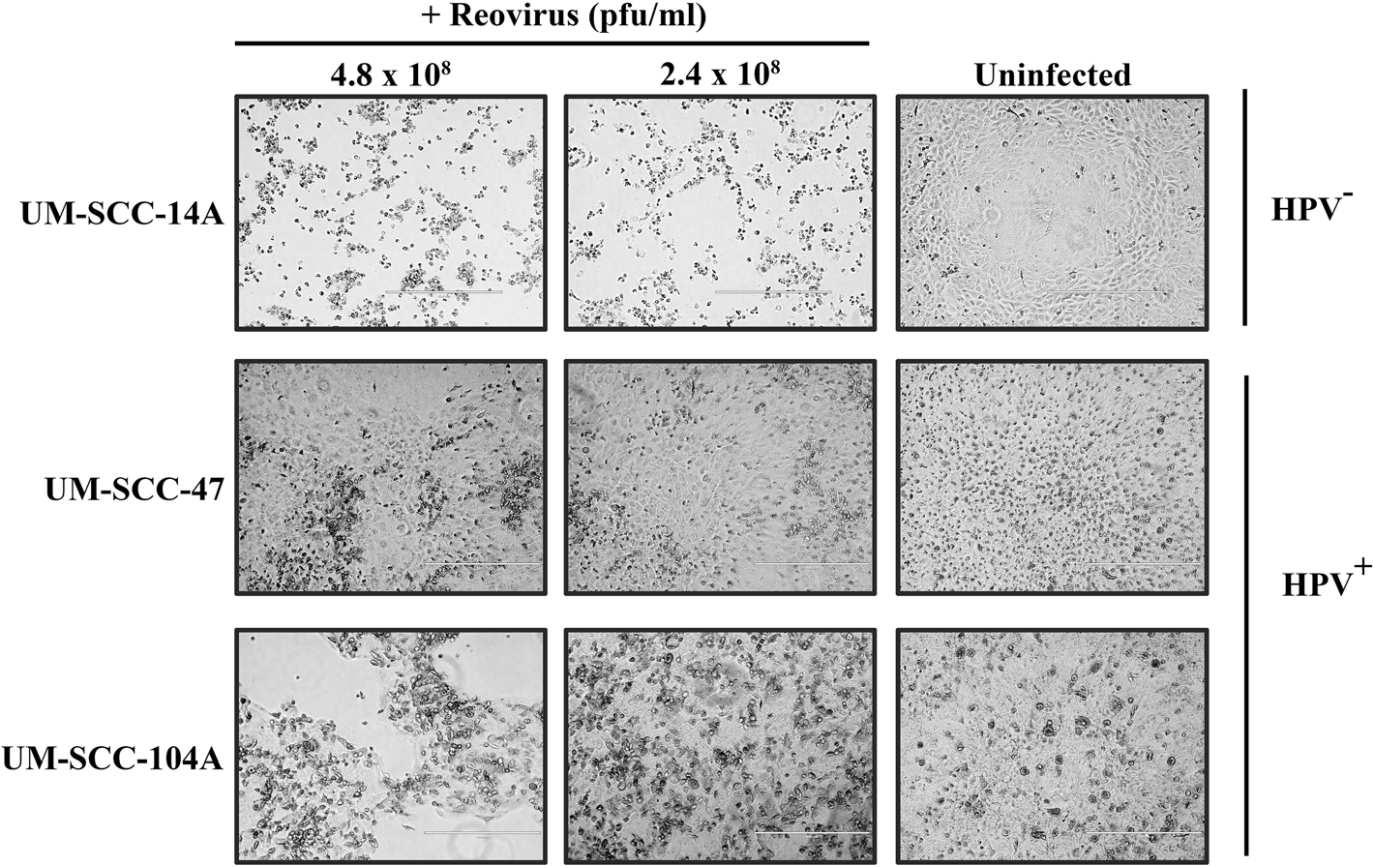
**Brightfield microscopy of UM-SCC-14A, UM-SCC-47, and UM-SCC-104 cells 96 h after the addition of 4.8×10** ^**8**^ **and 2.4×10** ^**8**^ **PFU/mL reovirus dilutions according to experiment protocol compared to uninfected controls**

Differences in percent viability were also found between cell lines 96 h after infection with reovirus at a concentration of 2.40x10^8^ PFU/mL. This equates to an MOI of 7.68×10^3^ viral particles per cell. The mean percent viabilities for each cell line from least to greatest were UM-SCC-14A (6.7 ± 5.0%), FaDU (10.9 ± 3.7%), SCC-9 (33.2 ± 9.9%), SCC-25 (54.6 ± 21.5%), UM-SCC-104 (82.3 ± 6.5%), UM-SCC-38 (83.9 ± 16.3%), and UM-SCC-47 (97.2 ± 4.7%) (Figure 2). The two most susceptible cell lines to virally-induced cytotoxicity were UM-SCC-14A and FaDU which were both HPV negative. Of the three cell lines with the greatest viability at this time point, two were HPV positive (UM-SCC-104 and UM-SCC-47). UM-SCC-47 had more viable cells with statistical significance than all of the HPV negative cell lines except for UM-SCC-38 (p = 0.17). UM-SCC-104 had more viable cells with statistical significance than UM-SCC-14A, FaDU, and SCC-9 (all with p < 0.01). The HPV positive cell lines were highly resistant to oncolysis by reovirus and showed only minimal viral-induced cytotoxicity at 96 h, even with high concentrations of reovirus used for infection. Images taken from brightfield microscopy at 96 hours after infection of the UM-SCC-14A, UM-SCC-47, and UM-SCC-104 cell lines demonstrate this difference (Figure 3).

### Oncolysis

**Figure 4:**
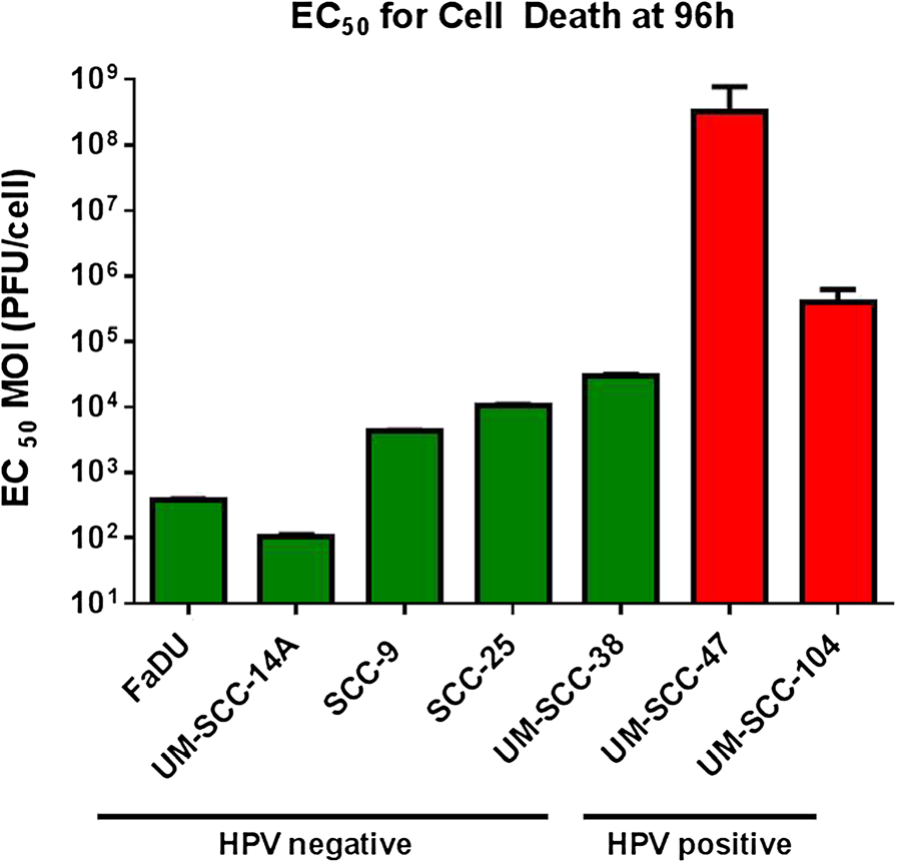
**EC**_**50**_ **values for oncolysis 96 h after reovirus infection of various HPV negative and positive HNSCC cell lines.** Error bars represent standard deviation.

The head and neck cancer cell lines had variable EC_50_ values for cell death at 96 h. The HNSCC cell line most susceptible to reovirus was UM-SCC-14A (HPV negative) with a mean EC_50_ MOI (PFU/cell) value of 102 (95%CI [93–112]). This means that 102 reovirus particles per cell were sufficient to cause 50% cell death in this cell line. The remaining cell lines from most to least susceptible to reovirus-mediated oncolysis and their corresponding EC_50_ MOI (PFU/cell) values were FaDU (388, CI[378–397]), SCC-9 (4.24×10^3^, CI[4.00×10^3^–4.49×10^3^]), SCC-25 (1.07×10^4^, CI[1.03×10^4^–1.10×10^4^]), UM-SCC-38 (2.99×10^4^, CI[2.80×10^4^–3.18×10^4^]), UM-SCC-104 (4.04×10^5^, CI[2.62×10^5^–6.23×10^5^]), and UM-SCC-47 (3.19×10^8^, CI[1.31×10^8^–7.76×10^8^) (Figure 4). The two HPV positive cell lines were more resistant to reovirus-mediated oncolysis in comparison to the HPV negative cell lines (p < 0.01 in all cases).

## Discussion

The use of viruses in cancer therapy is a rapidly expanding area of research [13,16,23,27,34]. However, the use of viral oncolytic therapy has yet to make the transition from bench to bedside in standard practice. Reovirus was first shown to have an oncolytic effect in head and neck cancer cells by Ikeda et al. [22] using *in vitro* and *in vivo* models. This effect has been demonstrated in numerous head and neck cell lines [24,25,36,37]. The oncolytic effect is believed to be independent of epidermal growth factor receptor (EGFR) activation and molecular predictors of response have yet to be identified [25]. Preclinical studies have shown the effectiveness of a combination of reovirus, paclitaxel and cisplatin in head and neck cancer lines [24]. Also, animal models have suggested a role for reovirus as an adjunct in surgically resected disease with positive margins [37]. Intravenously administered reovirus in combination with carboplatin and paclitaxel has been shown to have activity in advanced stage and recurrent head and neck cancer in a recently published phase I/II clinical trial [35]. An ongoing phase III trial is investigating intravenous reovirus in combination with paclitaxel and carboplatin (Reo 018).

Reovirus has variable infectivity and oncolytic activity in head and neck cancer cell lines and the mechanism behind this variable susceptibility has yet to be elucidated but is likely multifactorial. Our findings suggest an important difference in the susceptibility of head and neck cancer cells to reovirus based on HPV status. The HPV negative cell lines used were much more susceptible than the HPV positive cells to both infection by reovirus and virus-mediated oncolysis. There was a >150 fold difference in the amount of virus required to infect 50% of cells in the most susceptible cell line (SCC-9) and the least susceptible cell line (UM-SCC-104). Similarly, there was a dramatic difference between oncolysis based on HPV status. There was a 3x10^6^ fold difference in the EC_50_ values of the most susceptible cell line UM-SCC-14A (HPV negative) and the most resistant cell line UM-SCC-47 (HPV positive). For both infectivity at 18 h and oncolysis at 96 h, the HPV negative cells were more susceptible than the HPV positive cells by highly significant values. Our study is the first to compare the oncolytic activity of reovirus in HPV positive and negative head and neck cancer cell lines. Also, it is the first to compare reovirus infectivity among head and neck cancer cell lines.

HPV positive (vs negative) oropharyngeal squamous cell carcinoma (OPSCC) has been shown to have a more favourable response to treatment with surgical and non-surgical treatments [6,7]. However, when considering treatment with cetuximab, a monoclonal antibody that targets EGFR, a number of studies suggest HPV positive OPSCC tumors may be less responsive to this chemotherapeutic drug [43,44]. This is consistent with several studies showing an inverse relationship with HPV positivity [44]. It is important to note that both reovirus and cetuximab act on Ras-dependent pathways [44]. Taken together, our results showing resistance to reovirus in HPV-positive HNSCC cell lines could therefore be due to a lack of EGFR expression and its downstream Ras-dependent treatment response.

Novel therapies are needed in head and neck cancer, especially in patients with HPV negative malignancies. Conventional therapy is associated with substantial morbidity and long-term complications [1], and progress has been limited in the use of adjuvant therapy in patients with advanced stage HPV negative cancers [45]. Reovirus shows promise as a potential novel therapy in HPV negative head and neck cancer.

Further research is required to identify additional molecular markers for susceptibility to reovirus to identify patients most likely to benefit from adjunctive reovirus therapy. HPV negative patients, a group with a poor prognosis relative to those with HPV-related head and neck cancer, are identified as a group to target in future reovirus trials. Ongoing and future trials investigating reovirus in head and neck cancer may need to perform subgroup analysis based on HPV status.

Commonly described features of HNSCC cell lines include tumor subsite, staging, and treatment modalities utilized. Although clinically relevant, the smoking history of the patients from which these cell lines have been derived is not well described in the literature. The smoking status of the patients from which FaDU and SCC-9 were derived is not documented. The source of SCC-25 had an extensive history of smoking [46]. Of the cell lines obtained from Dr. Carey and the University of Michigan, UM-SCC-14A, UM-SCC-38, and UM-SCC-104 were derived from smokers [47]. However, there is no laboratory documentation regarding the smoking status of the patient from which the HPV positive UM-SCC-47 cell line was derived. Despite this limitation in clinical history, numerous papers have delineated genotypic differences between these and other HNSCC cell lines [48].

There are several limitations to this study. The behavior of cell lines in *in vitro* experiments is variable. Confounding factors between the cell lines used beyond HPV status may have an impact on results. Head and neck cancer is a molecularly and genetically heterogeneous entity [48,49]. Therefore, caution must be used in generalizing the effect of reovirus on a selection of cell lines to all HPV positive or negative head and neck cancers. However, this study design allowed for a time and cost efficient way to test a hypothesis regarding the activity of reovirus and HPV positive and negative head and neck cancers. Further investigation into the effect of reovirus on additional HPV positive and negative cell lines as well as in HPV positive and negative animal models is warranted.

## Conclusions

HPV negative cell lines appear to be more susceptible to reovirus infection and oncolysis than their HPV positive counterparts. Reovirus shows promise as a potential novel therapy in HPV negative head and neck cancer.

## Ethics approval

Prior to commencement, health research ethics board approval was obtained from the University of Alberta Health Research Ethics Board.

## Abbreviations

CsCl: Cesium chloride
EC_50_: Effective concentration 50%
EGFR: Epidermal growth factor receptor
HNSCC: Head and neck squamous cell carcinoma
HPV: Human papillomavirus
MgCl_2_: Magnesium chloride
MOI: Multiplicity of infection
OPSCC: Oropharyngeal squamous cell carcinoma
PBS: Phosphate buffered saline
PFU: Plaque forming units
RPM: Rotations per minute
